# Essay on Salivary Calculus

**Published:** 1842-09

**Authors:** 


*Essay on Salivary Calculus.*—We hope that the American Society of Dental Surgeons will lose no time in publishing the excellent essay read by Edward Maynard, M. D., at their late meeting in Boston, and we hope that the members generally will supply themselves with it. Dr. M. has treated the subject in such a manner as will hardly fail to convince all who read it of the deleterious effects which this substance exerts upon the gums, fluids of the mouth, and teeth. It should be in every family.

